# DanQi Pill protects against heart failure through the arachidonic acid metabolism pathway by attenuating different cyclooxygenases and leukotrienes B4

**DOI:** 10.1186/1472-6882-14-67

**Published:** 2014-02-20

**Authors:** Yong Wang, Chun Li, Zhongyang Liu, Tianjiao Shi, Qiyan Wang, Dong Li, Yan Wu, Jing Han, Shuzhen Guo, Binghua Tang, Wei Wang

**Affiliations:** 1 Beijing University of Chinese Medicine, Bei San Huan Dong Lu, Chao Yang district, Beijing, China; 2 Modern Research Center for Traditional Chinese Medicine, Beijing University of Chinese Medicine, Bei San Huan Dong Lu, Chao Yang district, Beijing, China; 3 State Key Laboratory of Proteomics, Beijing Proteome Research Center, Institute of Radiation Medicine, Science Park Road, Beijing, China

## Abstract

**Background:**

Chinese herbal formulae are composed of complex components and produce comprehensive pharmacological effects. Unlike chemical drugs that have only one clear single target, the components of Chinese herbal formulae have multiple channels and targets. How to discover the pharmacological targets of Chinese herbal formulae and their underlying molecular mechanism are still under investigation.

**Methods:**

DanQi pill (DQP), which is one of the widely prescribed traditional Chinese medicines, is applied as an example drug. In this study, we used the drug target prediction model (DrugCIPHER-CS) to examine the underlying molecular mechanism of DQP, followed by experimental validation.

**Results:**

A novel therapeutic effect pattern of DQP was identified. After determining the compounds in DQP, we used DrugCIPHER-CS to predict their potential targets. These potential targets were significantly enriched in well-known cardiovascular disease-related pathways. For example, the biological processes of neuroactive ligand–receptor interaction, calcium-signaling pathway, and aminoacyl–tRNA biosynthesis were involved. A new and significant pathway, arachidonic acid (AA) metabolism, was also identified in this study. This predicted pathway alteration was validated with an animal model of heart failure (HF). Results show that DQP had effect both on thromboxane B2 (TXB2) and Prostaglandin I2 (PGI2) in different patterns. It can down-regulate the TXB2 and up-regulate the PGI2 in diverse way. Remarkably, it also had effect on cyclooxygenase (COX)-1 and COX2 by suppressing their levels, which may be the critical and novel mechanism of cardiacprotective efficacy for DQP. Furthermore, leukotrienes B4 (LTB4) receptor, another key molecule of AA metabolism which finally mediated gastrotoxic leukotrienes, was also reduced by DQP.

**Conclusions:**

The combination of drug target prediction and experimental validation provides new insights into the complicated mechanism of DQP.

## Background

Heart failure (HF) after myocardial infarction (MI) is a common clinical syndrome with high morbidity and mortality [1]. Although traditional risk factors partially account for the development of HF, other new factors have recently been implicated. Evidence suggests that inflammation is important in the development and progression of HF. Chronic heart failure (HF) is characterized by an ongoing inflammatory response that correlates with HF disease severity and prognosis [2]. Given the importance of inflammatory pathways in HF, new treatment modalities that target inflammatory mediators may be effective. Recently, a series of experimental studies has reported the improvement of myocardial infraction and HF through anti-arachidonic acid (AA) metabolism therapy, specifically by suppressing cyclooxygenase 1 (COX1) and COX2 [3]. However, these drugs have side effects, such as increased gastrointestinal and adverse cardiovascular events [4].

Traditional Chinese medicine has been applied to treat HF for thousands of years, and some herbal formulas have been proved to be effective [5]. Among them, the ancient DanQi Pill (DQP), prepared from a basic formula of two Chinese herbs (i.e., Salvia miltiorrhiza and Panax notoginseng), is widely produced in China in accordance with the China Pharmacopoeia standard of quality control [6]. DQP is commonly prescribed in routine treatment of HF in China. Large-scale randomized and controlled clinical trials have proved that DQP has a definite effect in improving heart function [7]. Until now, many studies are being conducted to investigate the effect of active monomers in DQP on HF. For example, panax notoginseng saponins (PNS, monomer of P. notoginseng) were found to affect ischemia-induced myocardial apoptosis [8]; tanshinone IIA (monomer of S. miltiorrhiza bunge) was found to have cardioprotective effects and to attenuate myocardial hypertrophy [9]. However, most of these studies examined only one or several pharmacological effects of the monomer. Studies on the comprehensive effects of all of the compounds are rare.

In recent years, researchers have developed some bioinformatic methods to infer drug–target interactions [10-15], which provide opportunities to conduct more efficient research on the efficacy of the entire formula. Recent advances on the databases cataloguing chemical components of herbs and the interactions between drugs and targets have contributed to predicting the drug targets of herbs.

DrugCIPHER-CS is an efficient drug–target prediction method that was successfully applied to our recent work [16,17]. In the current paper, we investigated the pharmacological mechanism of DQP using DrugCIPHER-CS to predict drug targets, followed by experimental validation. Specifically, after obtaining the potential targets of DQP, the significantly enriched Kyoto Encyclopedia of Genes and Genomes (KEGG) biological pathways and Gene Ontology (GO) biological processes involved in these potential targets were analyzed. AA metabolism pathway was selected to elucidate the special efficacy patterns of DQP. This study applied novel and effective methods and provided insight into the complicated multi-targets mechanism of herbs.

## Methods

### Drug–target prediction and analyses

We used DrugCIPHER-CS to predict the drug targets of the main compounds of DQP, following the procedure described in [17]. The herbal compounds were first identified in the Modern Chinese materia medica database [18]. The identified compounds were then typed into the durgbank (http://www.drugbank.ca/) to search their structural information including their canonical and isomeric SMILES. Then we applied DrugCIPHER-CS to predict the potential drug targets of these compounds. DrugCIPHER-CS achieved good prediction performance in our previous study and can infer drug-targets in the genome-wide scale [17]. This method is based on the hypotheses that i) drugs with similar chemical structure usually bind functionally related proteins, and ii) functional relationship between the proteins can be measured by their distance in the protein interaction network. Given a set of known drug (drug space)-target (target space) interactions, for a query drug and a candidate target-gene, drugCIPHER-CS will measure the likelihood of their interaction based on the correlation between the query drug’s structure similarity vector with the drug space and the candidate gene’s functional similarity vector with the target space. For a query compound, drugCIPHER-CS will prioritize the proteins in the protein interaction network (i.e. candidate proteins) according to the order of the decreasing drug-target interaction likelihood, and the candidate proteins with high likelihood will be hypothesized as the potential drug-targets [16].

Here known drug-target interactions are obtained from DrugBank database [19]. We only use those drug-target interactions whose drugs are FDA-approved and have InChI identifiers [20]. The chemical structure similarity is calculated based on compounds’ MOLPRINT 2D descriptors and Tanimoto coefficient [21].

After obtaining the potential drug targets, we analyzed the significantly enriched KEGG biological pathways and GO biological processes of these potential targets using the hypergeometric cumulative distribution test [22]. A pathway was significantly enriched with candidate target-genes when its corresponding upper-tailed P-value of hypergeometric cumulative distribution was smaller than 0.05. The pathways were ranked according to the order of the increasing P-values. Then, GO annotations of human proteins were obtained from the GO project Web site (http://www.geneontology.org/) [23]. The top 0.1% candidate target-genes were significantly enriched with genes annotated with a GO term when its corresponding upper-tailed P-value of hypergeometric cumulative distribution was smaller than 0.05. These GO terms were ranked according to the order of the increasing P-values. KEGG biological pathway data were downloaded from the KEGG database [24].

### HF model preparation and grouping

Studies were performed in accordance with the China Physiological Society’s “Guiding Principles in the Care and Use of Animals” and with approval of the Animal Care Committee of Beijing Medical Center (SCXK: 2006-0009). A total of 80 male Specific Pathogen Free (SPF)-grade Sprague–Dawley (SD) rats weighing 240 ± 10 g were selected (purchased from Beijing Vital River Laboratory Animal Technology Co. Ltd.). HF was induced by direct coronary ligation as previously described [25]. Briefly, sixty SD rats were anaesthetized with pentobarbital sodium (1%, 50 mg kg − 1 intraperitoneally). The left anterior descending coronary artery (LAD) was ligated with a 5–0 polypropylene suture (Surgipro, CT, USA) directly proximal to its main branching point. After electrocardiograph testing, rats that averaged QT-interval prolongation in three pre-cordial leads were included in the study. Sham-operated groups of the rest twenty rats were prepared following an identical procedure, but without the actual tying of the polypropylene suture. The overall mortality of rats that underwent induction of HF during the entire experimental period (up to 28 days after operation) was 30%. The majority of death occurred on the day of or the day after the surgery, probably because of acute pump failure or lethal arrhythmias. The operated rats were then randomly divided into two groups: 20 in the model group, 20 in the DQP group. Meanwhile, 20 in sham-operated group were investigated together. The rats were fed with a standard diet and water, and were maintained on a 12 h light and dark cycle. The DQP group was treated for 28 days, with the total daily dosage of 1.5 g/kg of the concentrated DQP (Tongren Tang, Beijing, China) dissolved in water. The sham and model groups received the same volume of water through oral gavage as the DQP vehicle. At the end of the study, all animals were anaesthetized using pentobarbital sodium following an overnight fast. Blood samples were collected through abdominal aorta puncture and were placing on ice. After centrifugation, plasma was collected, aliquoted, and stored at -80°C until analysis of each indicator. The heart was excised and incubated in ice-cold PBS to wash out blood. Each left ventricle was then carefully dissected to remove all the necrotic/scarred zones to keep only the viable myocardium in the marginal zone of the infarct region in model animals. The left ventricular myocardial below ligation bit in sham animals were also dissected.

### Echocardiographic assessment of the left ventricle (LV) function

Echocardiography was used to detect left ventricle (LV) end-systolic diameter (LVEDs), LV end-diastolic diameter (LVEDd), ejection fraction (EF) and fractional shortening (FS). A sector scanner (PST 65A, Toshiba Company, Japan), which generates two-dimensional images at a frame rate of 300 frames/s to 500 frames/s, was used. Fractional shortening (FS%) was calculated by the following equation: FS% = [( LVEDd- LVEDs)/LVEDd]*100%.

### Preparation and dose consideration of concentrated DQP

The DQP (Series: 6128006) used in this study was manufactured by Tongrentang company (Beijing, China) with the roots of 150 g S. miltiorrhiza and 150 g P. notoginseng. Briefly, the residue of P. notoginseng was mixed with all S. miltiorrhiza bunge, followed by extraction with hot water (twice at 2 h each). The water extract was then concentrated to form a paste, and ethanol was added. After 24 h, the filtrate, which was the final product, was collected. Based on the recommendation of daily human dosage (20 g/d) and the equivalent conversion between animal and people by body surface area and body weight, dosage of 1.5 g/kg was chosen in the present study.

### Measurement of plasma indicators by Enzyme Linked Immunosorbent Assay (ELISA)

Levels of plasma indicators were quantified in duplicate using commercial ELISA kits (Abcam Inc., Cambridge, MA, USA). Each assay was performed following the kits’ instructions. Standards at a series of concentrations were run parallel to the samples. The concentrations of the samples were calculated in reference to their corresponding standard curves and expressed as ng/mL.

### Measurement of indicators by Western blot

The heart tissue was homogenized in RIPALYSIS buffer (i.e., 50 mM Tris-HCl pH7.4, 150 mM NaCl, 2 mM EDTA, 1% NP-40, and 0.1% SDS), and total protein was extracted from this homogenate. The protein concentration in each extract was measured using a protein assay kit (Pierce; Rockford, IL) and was then adjusted to the same value in all samples with 2× 4% Sodium dodecyl sulfate (SDS) sample buffer. The samples were boiled for 5 min, followed by loading on a 12.5% SDS-Polyacrylamide gel electrophoresis (PAGE) gel (30 mg protein/10 ml per well) for electrophoresis using a Bio-Rad mini gel apparatus at 100 V for 2 h. The fractionated protein on the gel was transferred onto an NC membrane (Millipore) and electrophoresed at 300 mA for 90 min. The membrane was first probed with COX1 primary antibody (anti-COX Type 1 antibody, ab18801, Abcam, 1:500) and secondary antibody (donkey polyclonal secondary antibody to rabbit IgG-HRP,ab97064, Abcam, 1:5000), and then treated with ECL (ECL plus Western blotting detection reagent, GE Healthcare) for 1 min at room temperature. The bands in the membrane were visualized and analyzed using UVP BioImaging Systems. After obtaining the COX1 blot density, the membrane was then treated using Restore Western Blot Stripping Buffer (Thermo Scientific) to remove the COX1signal, followed by probing with glyceraldehyde-3-phosphate dehydrogenase (GAPDH) primary antibodies (GAPDH mouse monoclonal IgG, ab8245, Abcam, 1:2000). The COX2, prostaglandin E2 receptor 4 (EP4), and leukotrienes B4 receptor (LTB4R) antibody blot densities were determined using the same process. The final reported data were the normalized COX1 band densities by GAPDH.

### Statistical analysis

ANOVA using SAS 9.2 statistical software (SAS Institute, NC, USA) was applied to evaluate between-group differences in the outcome variables, follow-up least significant differences (LSD) analysis verified these differences were significant. P < 0.05 was considered statistically significant. Results were presented as mean values with their corresponding standard deviations.

## Results

### Drug target prediction and analyses

Through a comprehensive search, we collected 92 compounds of DQP with 50 and 42 in Danshen and Sanqi respectively. The DrugCIPHER-CS method [17] was used to infer the potential targets of the compounds (see Methods). For each of the compound of DQP, DrugCIPHER-CS ranked its candidate targets according to the order of decreasing possibility of being targeted by the compound. We chose the top 0.1% candidate targets of each compound and consequently obtained 232 candidate target genes. These candidate targets were significantly enriched as known cardiovascular disease-related genes (i.e., the known targets of drugs whose ACT code uses “C” as the first level) in DrugBank [16], consistent with the curative effect of DQP on cardiovascular disease.

Furthermore, we analyzed the enriched KEGG biological pathways among these potential targets. Seven significantly enriched pathways among the top 0.1% candidate targets were analyzed, including the pathways of neuroactive ligand–receptor interaction, AA metabolism, calcium-signaling pathway, aminoacyl-tRNA biosynthesis and renin-angiotensin system (RAAS) et al. (Table 1). These significantly enriched biological pathways provided important information on the mechanism of DQP. Many of these enriched pathways have been associated with HF, e.g., the importance of neuroactive ligand–receptor interaction in the development and progress of cardiovascular diseases [26]. The key proteins in this pathway, such as adrenergic, angiotensin, and calcitonin receptor-like neurotensin receptors, are closely related to cardiac function [27-29]. Aminoacyl-tRNA biosynthesis pathway is important in cardiovascular angiogenesis [30]. The relationship between calcium-signaling pathway and HF was also identified in the present study. Calcium antagonists have been widely used to inhibit extracellular calcium influx, reduce the concentration of intracellular calcium, and lower myocardial contractility [31]. Upregulation of the renin–angiotensin system is crucial in the deterioration of cardiovascular function [32]. However, the AA metabolism was rarely investigated both in HF and in DQP’s efficacy, it showed a significant difference as well as the pathway coverage in our study.

**Table 1:** Significantly enriched KEGG biological pathways among top 0.1% candidate target-genes of DQP compositive compounds

**KEGG pathway ID and name**	***P*-value**^**a**^	**Coverage (%)**^**b**^
Path:hsa04080 Neuroactive ligand-receptor interaction	2.66E-07	6.29
Path:hsa04020 calcium signaling pathway	5.22E-06	7.30
Path:hsa04614 renin-angiotensin system	5.64E-06	9.41
Path:hsa00970 aminoacyl-tRNA biosynthesis	4.67E-05	14.63
Path:hsa00250 alanine, aspartate and glutamate metabolism	1.29E-04	16.13
Path:hsa00590 arachidonic acid metabolism	2.46E-03	18.62
Path:hsa00330 arginine and proline metabolism	1.15E-02	7.41

^a^A pathway is significantly enriched with candidate target-genes when its corresponding upper-tailed P-value of hypergeometric cumulative distribution is smaller than 0.05. The pathways are ranked according to the order of the increasing P-values. ^b^The coverage for each pathway is referred to as the fraction of candidate target-genes among all the pathway member genes.

We also analyzed the functional distribution of these candidate targets (Table 2). The significantly enriched GO biological processes of these targets are cellular amino acid metabolic process, biosynthetic process, small molecule metabolic process, cellular nitrogen compound metabolic process, and circulatory system process, which suggest that the DQP may intervene in these progresses. These enriched pathways and GO functional annotations provided important clues to the molecular mechanism of DQP.

**Table 2:** Significantly enriched GO biological processes among top 0.1% candidate target-genes of DQP compositive compounds

**GO term ID**	**Description**	***P*-value**^**a**^
GO:0009058	Biosynthetic process	9.37E-09
GO:0006520	Cellular amino acid metabolic process	3.27E-08
GO:0034641	Cellular nitrogen compound metabolic process	3.56E-07
GO:0071941	Nitrogen cycle metabolic process	1.53E-05
GO:0006399	tRNA metabolic process	7.32E-05
GO:0044281	Small molecule metabolic process	1.16E-04
GO:0003013	Circulatory system process	1.43E-03
GO:0007267	Cell-cell signaling	3.33E-03
GO:0006950	Response to stress	3.63E-03
GO:0006412	Translation	9.13E-03
GO:0042592	Homeostatic process	1.19E-02
GO:0007568	Aging	1.60E-02
GO:0050877	Neurological system process	1.63E-02
GO:0008283	Cell proliferation	2.55E-02
GO:0008219	Cell death	4.09E-02

^a^The top 0.1% candidate target-genes are significantly enriched with genes annotated with a GO term when its corresponding upper-tailed P-value of hypergeometric cumulative distribution is smaller than 0.05. These GO terms are ranked according to the order of the increasing P-values.

### Model evaluation

**Figure 1:**
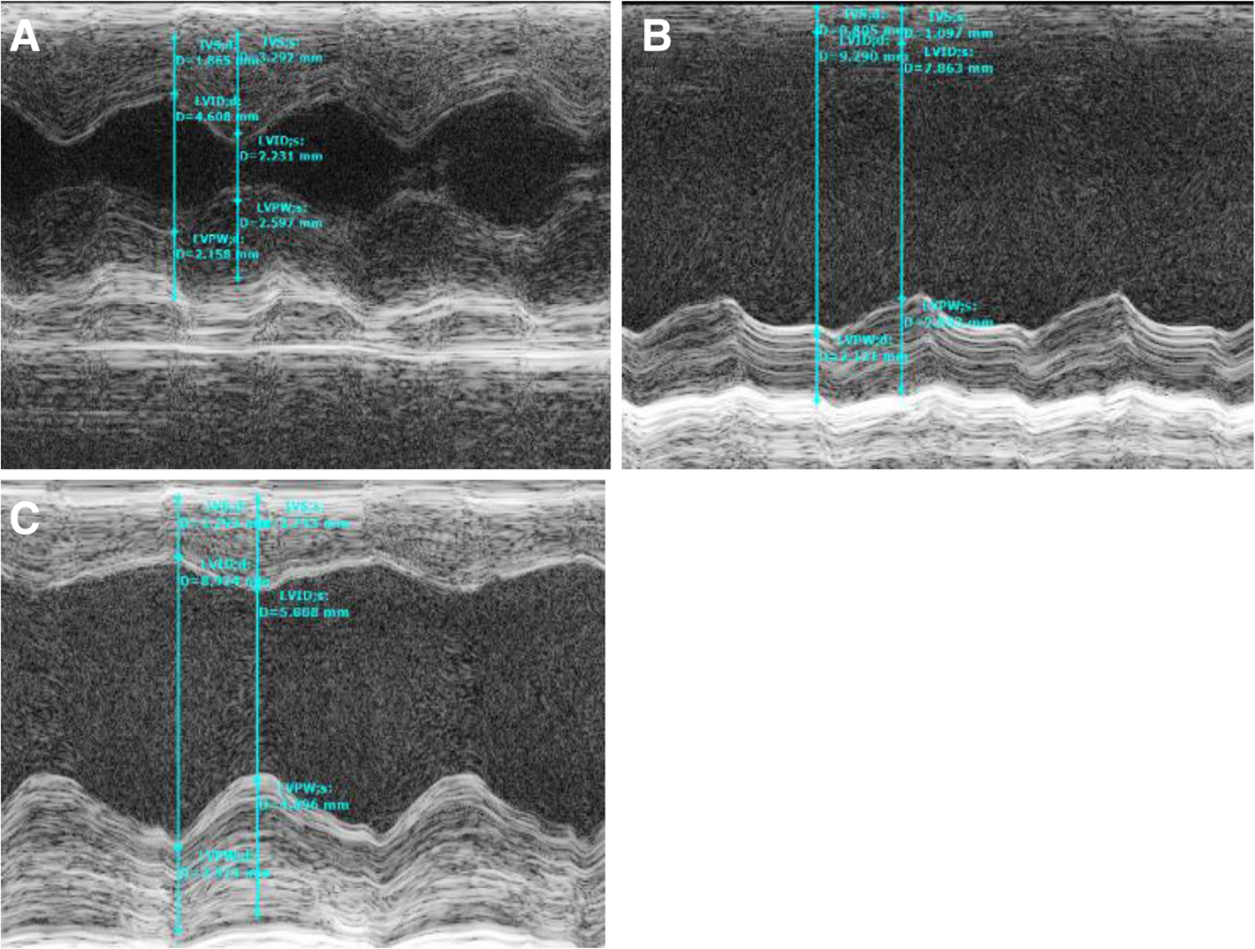
**The cardiac function in different groups.** Cardiac function detected by echocardiography. **(A)** Normal cardiac function including LVEF and LVFS in sham-operated group. **(B)** Down-regulation of LVEF and LVFS in model group rats. **(C)** DQP can significantly up-regulate the EF and FS. **A**: sham group; **B**. model group; **C**. DQP group.

Twenty-eight days after surgery, the EF and FS values of rats in the model group were significantly lower than those of the sham group, as indicated by echocardiography (P < 0.05, Figure 1). EF values of ligation rats in model group dropped compared with those of the sham group, which suggested that a steady HF model was established. Twenty-eight days after treatment with DQP, EF values in the treatment group increased compared with those in the model group (Figure 1, Table 3).

**Table 3:** Echocardiographic changes observed in different groups (x ± s)

**Group**	**Sham**	**Model**	**DQP**	***P *** **value**
**LVEDd (cm)**	0.74 ± 0.047^**^	1.05 ± 0.107^▲▲^	0.95 ± 0.128^▲▲^	0.001
**LVEDs (cm)**	0.35 ± 0.055^**^	0.85 ± 0.078^▲▲^	0.70 ± 0.203^▲▲*^	0.000
**FS**	52.47 ± 5.423^**^	18.71 ± 1.675^▲▲^	28.07 ± 11.879^▲▲^	0.025
**EF**	87.41 ± 4.271^**^	42.86 ± 3.110^▲▲^	57.38 ± 18.519^▲▲**^	0.017

Cardiac function was detected by echocardiography. Normal cardiac function was found in sham-operated group including LVEDd, LVEDs, LVEF and LVFS. Down-regulation of cardiac function appeared in model group rats. DQP can significantly up-regulate the EF and FS, and reduced the LVEDs. ^▲▲^P < 0.01, vs sham- operated group; ^*^P < 0.05, ^**^P < 0.01, vs model group.

### Effects of DQP on myocardial fibrosis

**Figure 2:**
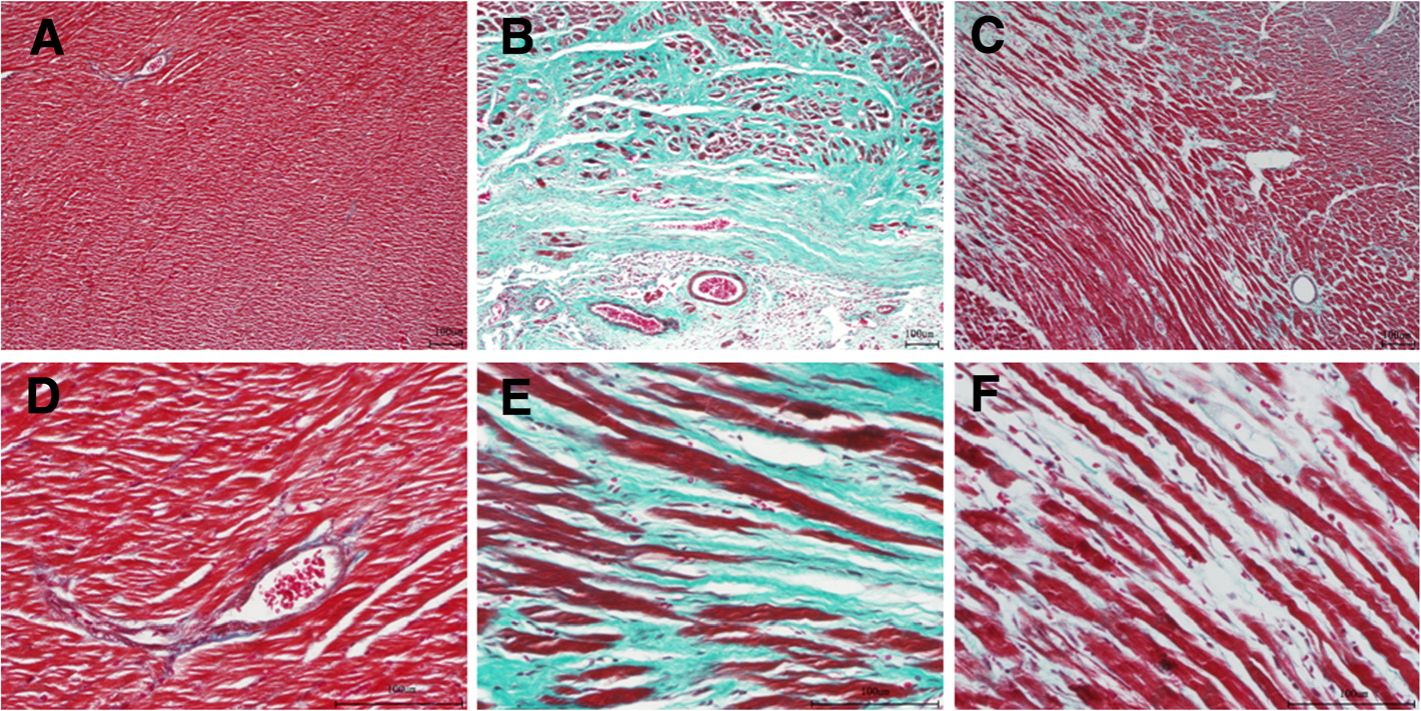
**Masson results in different groups. (A)** Cardiomyocytes in the sham group were orderly arranged. **(B)** Thickening and lengthening of myocardial fibers were observed in the model group. The nuclei were stained dark, which indicated local tissue fibrosis. **(C)** DQP had the inhibitory effect on ventricular hypertrophy. **A**: sham group (×100); **B**. model group (×100); **C**. DQP group (×100); **D**: sham group (×400); **E**. model group (×400); **F**. DQP group (×400).

The MASSON images of the left ventricular tissue were shown in Figure 2. Cardiomyocytes in the sham group were orderly arranged. Thickening and lengthening of myocardial fibers were observed in the model group. The nuclei were stained dark, which indicated local tissue fibrosis in the model group, while DQP had the inhibitory effect on ventricular hypertrophy.

### Validation of the predicted target pathways

Neurohormonal activation is important in the development of cardiovascular disease such as HF. However, the role of AA metabolism in HF development is still not clear [33]. Some studies show that inflammatory cells can release AA, which may be further metabolized into active products mediating cardiac fibrosis and HF. Some of the metabolic products, such as prostacyclin, are considered potential therapeutic targets of HF [34]. In the current study, the alteration of the AA metabolism pathway in HF was identified by DrugCIPHER-CS prediction and validated in animal models.

**Figure 3:**
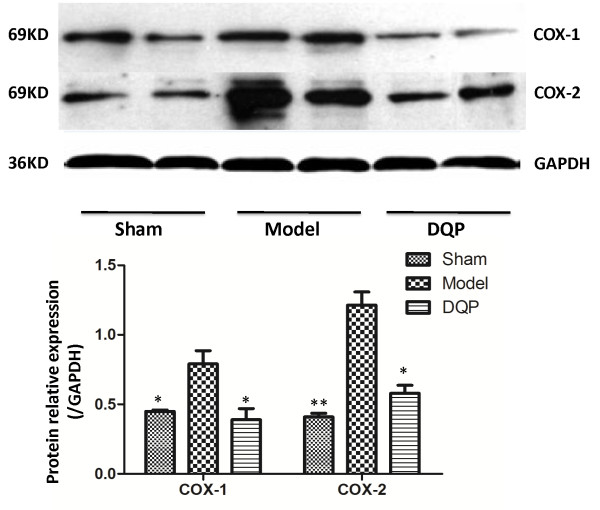
**DQP regulates COX1 and COX2 in HF rats significantly.** The results showed that in model group, COX1 and COX2 concentration was significantly higher than that in sham-operated group. In DQP group, level of COX1 and COX2 were decreased significantly. *P < 0.05, **P < 0.01, vs model group.

Western blot results showed that COX1 level in the model group (0.79 ± 0.096) increased 28 days after operation compared with that in the sham group (0.45 ± 0.01) (P = 0.031, Figure 3). After treatment with DQP, COX1 level in the DQP group (0.39 ± 0.08) decreased compared with that in the model group (P = 0.017). COX1 level in DQP-treated group showed no significant difference compared with that in the sham group (P = 0.896). COX2 level in the model group (1.213 ± 0.095) increased compared with that the sham group (0.409 ± 0.027) (P = 0.000). The level of COX2 in the DQP group (0.58 ± 0.057) decreased compared with that in the model group (P = 0.011) 28 days after treatment, recovering almost to sham group level.

**Figure 4:**
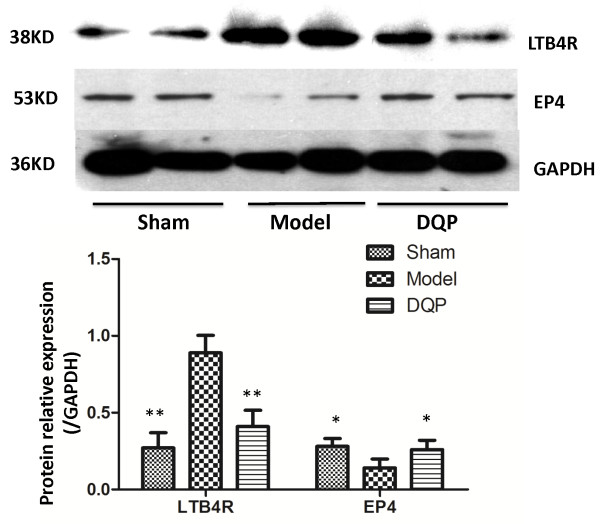
**Westernblot results of LTB4R and PGE2 receptor in different groups.** The results showed that in model group, LTB4R concentration significantly increased than that in sham-operated group. In DQP group, it was decreased significantly. While PGE2 receptor-EP4, in the model group decreased compared with that in the sham-operated group, and it was up-regulated by DQP in DQP group*.* *P < 0.05, **P < 0.01, vs model group.

Furthermore, LTB4 receptor (LTB4R) level in the model group (0.89 ± 0.113) was upregulated compared with that in the sham group (0.271 ± 0.098) (P = 0.000). Twenty-eight days after treatment with DQP, LTB4R level in the DQP-treated group (0.41 ± 0.106) decreased significantly compared with that in the model group (P =0.003, Figure 4).

Prostaglandin E2 (PGE2) and its receptors are considered better targets with protective effects in curing HF. Especially receptor 4 of PGE2, E-series prostanoid receptor 4 (EP4), is currently emerging as most versatile and promising among PGE2 receptors, and the PGE2/EP4 signalling is thought to be the potential targets to HF and myocardial ischemia [35]. In our study, EP4 of model group (0.14 ± 0.058) was down-regulated compared with that in the sham group (0.28 ± 0.052) (P = 0.023). In the DQP group, the EP4 level (0.26 ± 0.061) increased compared with that in the model group (P = 0.031), which had no significant difference with the sham group (Figure 4).

Prostacyclin I2 (PGI2) and thromboxane A2 (TXA2) are the products of AA metabolism through the COXs pathway. PGI2 has a cardioprotective effect by inhibiting platelet aggregation, whereas TXA2, which is quickly metabolized to TXB2, increases the risk of cardiovascular events [36]. In our study, ELISA results showed that levels of 6-Keto-PGF1α, which is the stable metabolite of PGI2, in the model group decreased compared with that in the sham group (P = 0.036). DQP upregulated 6-Keto-PGF1α levels in the model group almost back to normal conditions 28 days after administration. TXB2 is the product of TXA2 and can therefore reflect the level of TXA2 in plasma. The level of TXB2 increased in the model group. Although DQP showed no significant effect on TXB2, DQP increased the 6-Keto-PGF1α-to-TXB2 (P/T) ratio (Table 4).

**Table 4:** Levels of 6-Keto-PGF1α and TXB2 in different groups

**Group**	**Sham**	**Model**	**DQP**	**P value**
**6-Keto-PGF1α (pg/ml)**	240.09 ± 77.365^*^	78.33 ± 39.043^▲^	230.89 ± 85.021^*^	0.016
**TXB2 (pg/ml)**	88.53 ± 9.139^*^	271.01 ± 156.103^▲^	168.31 ± 104.670	0.033
**Ratio of P/T**	1.81 ± 0.839^*^	0.81 ± 0.481^▲^	1.47 ± 0.688^*^	0.024

The results showed that in model group, concentration of TXB2 significantly increased than that in sham-operated group. In DQP group, it was decreased. 6-Keto-PGF1α in the model group decreased compared with that in the sham-operated group, and it can be up-regulated by DQP*.* *Mean values were significantly different from model (*P < 0.05), ^▲^Mean values were significantly different from sham (^▲^P < 0.05).

## Discussion

In the present study, drug target prediction was applied to reveal the underlying mechanism of DQP. A potential target pathway seldom studied, AA metabolism, was investigated. After experimental validation, COXs and LTB4 were determined to be the targets of DQP.

Both nonselective and COX-specific inhibitors called nonsteroidal anti-inflammatory drugs are commonly used in relieving pain associated with medical conditions [37]. COX (prostaglandin G/H synthase) is a key enzyme in prostaglandin synthesis; it has two isoforms (i.e., COX1 and COX2) [38]. COX1 is constitutively expressed and is probably responsible for prostaglandin release under physiological conditions, and COX2 is expressed at high levels upon induction. COX activation produces prostaglandin H2, which is subsequently converted to PGE2, PGI2, or thromboxane A2 (TXA2). Then, the PGI2 and TXA2 are unstable in plasma, and quickly metabolized to 6-Keto-PGF1α and TXB2 respectively [39]. Imbalance between TXA2 and PGI2 is considered as a critical cause of HF. Anti-COXs therapy with aspirin, which at low doses acts as a selective inhibitor of COX activity, is well established. However, a major limitation of aspirin treatment is its gastrointestinal toxicity, which is considered linked to its disruption of the balance between TXA2 and PGI2. Newly developed dual COX2 and LTB4 inhibitors not only share the anti-inflammatory effect of COX1 inhibitors but also inhibit the 5-lipoxygenase (LOX)-mediated synthesis of gastrotoxic leukotrienes. Dual inhibitors may be beneficial in the treatment of HF with fewer side effects [40].

In the present study, using DrugCipher-CS software, AA metabolism was predicted to be altered by DQP. An experiment using an animal model validated that the alteration of the levels of proteins in the AA metabolism pathway with the occurrence of HF and administration of DQP to HF rats could suppress these protein levels. Consistent with previous reports, the LAD-induced HF animals were characterized by declined diastolic and systolic myocardial performance (i.e., decreased EF and FS values) and myocardial fibrosis. Our results also agree with those of previous studies that demonstrate the enhanced activation of cardiac AA metabolism in HF, such as PGI2 and TXB2 [41]. Moreover, COXs and LTB4 are the products of two AA metabolism pathways mediated by COX and LOX, respectively. Levels of COXs and LTB4R increased in HF models because of the activation of AA metabolism.

DQP treatment was able to regulate the AA metabolism in different ways. First, COX1 and COX2 are downregulated, thus suppressing AA activation and avoiding its gastrointestinal toxicity. Downregulation of LTB4R also occurs. LTB4 produced from AA by 5-LOX is a potent chemo-attractant of leukocytes [42]. Therefore, the cardioprotective effect on HF by DQP is partly due to its inhibition of LTB4R. These results also support the emerging role of inflammation in the development and progression of HF [33,43].

PGI2 and TXA2 belong to metabolites of AA pathway, and they have critical and contradictory roles in the progression of HF. PGI2 exhibits cardioprotective effects by inhibiting the aggravation of HF. However, TXA2, which is metabolized into TXB2, increases the risk of cardiovascular events. The dynamic balance between the metabolites is considered a critical biomarker of thrombosis regulation in HF [44]. Although DQP cannot directly reduce the concentration of TXB2, DQP can significantly upregulate the cardioprotective PGI2 and recover the imbalance of P/T ratio, thus providing a synthetic effect on HF.

## Conclusions

Our results suggest that DQP exerts a synthetic cardiac protective role by targeting multiple targets in the treatment of HF. Particularly, DQP can attenuate the activation of AA metabolism in HF by reducing the levels of COXs and LTB4R while increasing the levels of PGE2 receptor EP4 and PGI2. The combination of drug target prediction and experimental validation provides new insights into the complicated mechanism of DQP in the treatment of HF.

### Limitations

There are some limitations in our study. For example, the search for DQP compounds has been done through the Chinese Materia Medica only, which is far from comprehensive. We presume all components of herbal formulation compounds are absorbed and utilized; improvement should be made in our future work.

## Competing interests

The authors declare that they have no competing interests.

## Authors’ contributions

Conceived and designed the experiments: WW and BT. Performed the experiments: YW, CL and TS. Analyzed the data: ZL, DL and SG. Contributed reagents/materials/analysis tools: YW, JH. Wrote the paper: YW, ZL and QW. All authors read and approved the final manuscript.

## Pre-publication history

The pre-publication history for this paper can be accessed here:

http://www.biomedcentral.com/1472-6882/14/67/prepub
